# Global health and national borders: the ethics of foreign aid in a time of financial crisis

**DOI:** 10.1186/1744-8603-8-19

**Published:** 2012-06-28

**Authors:** Mira Johri, Ryoa Chung, Angus Dawson, Ted Schrecker

**Affiliations:** 1 Unité de Santé Internationale (USI), Centre de Recherche du Centre Hospitalier de l’Université de Montréal (CRCHUM), Édifice St-Urbain 3875, rue St-Urbain 5e étage, Montréal, QC, H2W 1V1, Canada; 2 Department of Philosophy, Faculty of Arts and Science, University of Montreal, Pavillon 2910 Édouard-Montpetit, 2910, boul. Édouard-Montpetit, Montréal, QC, H3T 1J7, Canada; 3 School of Health and Population Sciences, University of Birmingham, Edgbaston, Birmingham, B15 2TT, UK; 4 Institut de recherche Bruyère Research Institute 43, rue Bruyère St., Room 737D, Ottawa, ON, K1N 5C8, Canada

**Keywords:** Developing countries, Ethics, International Agencies, International Cooperation, Voluntary Health Agencies, World Health

## Abstract

**Background:**

The governments and citizens of the developed nations are increasingly called upon to contribute financially to health initiatives outside their borders. Although international development assistance for health has grown rapidly over the last two decades, austerity measures related to the 2008 and 2011 global financial crises may impact negatively on aid expenditures. The competition between national priorities and foreign aid commitments raises important ethical questions for donor nations. This paper aims to foster individual reflection and public debate on donor responsibilities for global health.

**Methods:**

We undertook a critical review of contemporary accounts of justice. We selected theories that: (i) articulate important and widely held moral intuitions; (ii) have had extensive impact on debates about global justice; (iii) represent diverse approaches to moral reasoning; and (iv) present distinct stances on the normative importance of national borders. Due to space limitations we limit the discussion to four frameworks.

**Results:**

Consequentialist, relational, human rights, and social contract approaches were considered. Responsibilities to provide international assistance were seen as significant by all four theories and place limits on the scope of acceptable national autonomy. Among the range of potential aid foci, interventions for health enjoyed consistent prominence. The four theories concur that there are important ethical responsibilities to support initiatives to improve the health of the worst off worldwide, but offer different rationales for intervention and suggest different implicit limits on responsibilities.

**Conclusions:**

Despite significant theoretical disagreements, four influential accounts of justice offer important reasons to support many current initiatives to promote global health. Ethical argumentation can complement pragmatic reasons to support global health interventions and provide an important foundation to strengthen collective action.

## Background

In keeping with the vision of “a more peaceful, prosperous and just world” enshrined in the United Nations Millennium Development Goals (MDGs) [1], initiatives to improve global health and human development have proliferated over the last decade [2-5]. Although developing countries play the leading role, the success of these strategies depends critically on the participation of the citizens and governments of the donor nations (principally, the state members of the Group of Eight Countries (G8) and the European Union) through financial assistance and supportive policies. International development assistance for health (DAH) has enjoyed a special priority among donors in recent years [6]. Resources quadrupled from $5.6 billion in 1990 to $21.8 billion in 2007, and the rate of growth accelerated sharply after 2002 [6].

The future of global health financing is much more uncertain. The global financial crisis that began in 2008 has placed aid budgets under pressure [7]. Although DAH continued to expand between 2007 and 2010, the rate of growth slowed dramatically [8]. Competition among global health priorities may also have intensified. In 2010, world leaders endorsed an ambitious new scheme to reach the MDGs by the 2015 target date through a focus on the health of the most vulnerable women and children [9]. Yet, funding for international assistance for HIV and AIDS provided by donor governments declined by 10 per cent over the 2009–2010 period, marking the first time year-to-year support for HIV and AIDS has fallen in more than a decade [10].

It is too early to know what the 2011 Eurozone crisis will mean for global health funding; however, a slowdown in global growth [11] and fiscal austerity in Europe and elsewhere will almost certainly put additional downward pressure on meeting aid targets [12-14]. The United States Congress is now considering the first significant cuts in overseas aid in nearly two decades, on the order of $12 billion, or 20 per cent of the President’s request for 2012 [15,16].

The competition between national priorities and foreign aid commitments raises important ethical questions. For some, the motivation to support global health is based on a principle of universal solidarity among human beings [3]. However, for many, national borders delimit the prime locus of moral responsibility. The duty to alleviate suffering abroad is seen as discretionary, and distinctly secondary to domestic concerns. Two arguments dovetail to support this latter perspective. A realist conception of international relations suggests that the proper role of every national government is to represent and advance the interests of its own nation. Similarly, many ethicists hold that we have more important moral duties towards co-nationals, with whom we share a common past, the benefits and burdens of social cooperation, and a common destiny [17]. The view that “charity begins at home” may seem particularly salient in the current context of financial uncertainty and the prospect of a global economic recession.

To ensure that global health priorities receive adequate and stable funding it will be essential not only to demonstrate the effectiveness of interventions and programmes [8], but also to clarify the reasons for our commitment to this goal. Theories of justice offer sophisticated frameworks through which moral choices and responsibilities can be analysed. Through a non-technical introduction to a range of influential theories from the ethics literature, this paper aims to foster individual reflection and public debate on donor responsibilities for global health. We also hope to illustrate the value of this approach in clarifying policy commitments that can be widely upheld under conditions of reasonable moral pluralism [18].

## Methods

This article critically reviews several contemporary accounts of justice important in the Western canon. Study selection followed a three-part procedure balancing author expertise (MJ, RC, and AJD have PhDs in philosophy with specialisation in ethics; RC and AJD hold academic positions as ethicists; TS, a social scientist, has published extensively on health ethics, global justice and human rights) and validation by qualified peers. (1) The authors first established a list of four criteria to be satisfied. Individual theories should: (i) articulate important and widely held moral intuitions, and (ii) have had extensive impact on debates about global justice. Collectively, they should: (iii) represent diverse approaches to moral reasoning, and (iv) present distinct stances on the normative importance of national borders. (2) Authors next generated an inclusive list of candidate theories, and shortened it through application of these criteria. (3) Finally, results were validated and refined on two separate occasions by specialists in global public health, ethics, and political philosophy. Additional file 1 contains a detailed description of the procedure.

Due to space limitations we limit the discussion to four frameworks. As we shall show, each suggests different conclusions about the nature and extent of our obligations to promote global health. Each theory is open to objections, which we do not wish to minimise or ignore; nor do we wish to endorse any particular position. We focus instead on areas of agreement. Our claim is that all of these views will accord to global health a serious moral importance implying substantial responsibilities that generally are not satisfied by current efforts.

## Results

We reviewed four theories representing consequentialist (Singer), relational (Pogge), human rights (Shue), and social contract (Rawls) approaches. These theories represent a variety of views on the normative significance of national borders.

### Four theories of justice

Cosmopolitans view all human beings as belonging (at least, potentially) to a single community. We discuss the most radically cosmopolitan theory of justice first, working through to the conception most clearly favourable to foregrounding the normative significance of national borders. Table 1 provides an overview of the four theories, Table 2 presents common objections to each view, and Table 3 offers examples of the types of policies that could be supported by each approach [19].

**Table 1:** **Importance of the health of the global poor**^**1**^**on four accounts of justice**

	**Singer**	**Pogge**	**Shue**	**Rawls**
**Addressed to whom?**	Indvidual moral agents	Individuals & national governments	Individuals & national governments	National governments & their peoples
**National borders important?**	No	Possibly	Yes	Yes
**Key concepts**	Individuals have an obligation to prevent the occurrence of something significantly bad if they can do so at acceptable cost to themselves.	We have a duty not to cause severe harm for minor gain. This obligation remains equally valid if an agent is responsible for causing harm in a jurisdiction outside his or her national borders, and is independent of whether we should privilege obligations to compatriots.	Two basic rights – subsistence and security–constitute pre-conditions for the enjoyment and exercise of all other rights and freedoms. Liberal democratic states have a duty to adopt foreign policies consistent with these fundamental human rights.	Under an idealised form of social contract, representatives of free and equal societies would adopt 8 principles of governance that enable an ideal global community to live together over time in peace, harmony and mutual respect.
**Is health of the global poor important?**	Yes	Yes, under certain conditions	Yes, to a limited extent	Yes, if useful to achieve just political arrangements
**Why?**	The global rich can ameliorate the suffering of the global poor with little sacrifice to themselves.	The international community is in some instances causally implicated in the genesis and perpetuation of severe poverty and ill health worldwide.	In instances where national governments fail to protect basic rights, others have a duty to guarantee their fulfilment. The right to subsistence guarantees every person worldwide a decent chance at a long and healthy life.	The 8 principles include a duty to “assist other peoples living under unfavourable conditions that prevent their having a just or decent political and social regime.” Empirical evidence shows that population health contributes to just political arrangements.
**What kind of obligation?**^2^	Justice	Justice	Justice	Justice or charity^3^
**What is the extent of the obligation?**	Until suffering has been eliminated	Until causal responsibility for harm has been corrected and adequately compensated^4^	Until a basic minimum has been provided	Until the international community has enabled burdened societies to develop just political arrangements
**Which health-related strategies should be privileged?**	Poverty alleviation & action on other determinants of health	Examination of national policy coherence to avoid causing or contributing to harms abroad;	Examination of national policy coherence to avoid depriving or contributing to deprivation abroad;	Those that strengthen basic institutions to a minimally decent threshold, enabling further social development. Candidate strategies could (1) promote equality of opportunity (especially in education and training), e.g. through child health; (2) offer additional synergies for development, e.g. by focussing on the rights and fundamental interests of women.
	Provision of health care	Analysis of the effects of global institutions	Provision of aid to ensure subsistence rights^6^, including guarantees related to the social determinants of health and minimal preventive health care.	
		Institutional reforms to promote satisfaction of human rights^5^		

^1^ The World Bank defines poverty as “pronounced deprivation in well-being” comprising multiple dimensions such as low incomes and the inability to acquire the basic goods and services necessary for survival with dignity, low levels of health and education, poor access to clean water and sanitation, inadequate physical security, lack of voice, and insufficient capacity and opportunity to better one’s life. The global poor are poor in an absolute sense [20].

^2^ Each theory takes a position on the question of whether duties towards the health of those outside our borders are matters of “justice” or “charity”. Duties of justice are precise, owed to specifiable others, and can in principle be legally enforced, whereas duties of charity admit of discretion in relation to their nature, timing, and choice of beneficiary. Charitable duties are adopted through conscious choice and are not legally enforceable.

^3^ For Rawls, the duty to assist is a duty of justice under the principles of the Law of Peoples. Beyond the threshold of minimal decency, the duty to assist becomes charity.

^4^ According to Pogge, degree of responsibility is proportional to benefits reaped and is discharged when proportional compensation is made [21].

^5^ For Pogge, a guarantee of human rights aims to confer on all human beings worldwide “secure access” to “minimally adequate shares” of basic freedoms of participation, of food, drink, clothing, shelter, education and health care [22].

^6^ For Shue, minimal economic security, or subsistence, entails “unpolluted air, unpolluted water, adequate food, adequate clothing, adequate shelter, and minimal preventive public health care [23].

**Table 2:** **Common criticisms of the four theories**^**1**^

	**Criticisms**	**Rejoinders**
**Singer**^**2**^	Moral priorities should focus on local need, for reasons similar to those raised in relation to national borders.	Singer allows that *psychologically* it might make a difference whether an individual is in severe need in front of one’s eyes or in a far-away country, but that it makes no *moral* difference.
	Singer demands too much of individuals as there will always be further work to do to relieve suffering somewhere in the world. All of one’s time could be spent relieving suffering, potentially endangering one’s own well-being.	This is unlikely to pose a problem in practice. Singer’s recent work aims to define attainable standards for living an ethical life in a world that contains great affluence and extreme poverty [24].
	Any obligation to respond to the challenges of global health should be understood as one of charity rather than justice.	For Singer, the severity of the suffering involved means that talk of charity is inappropriate. Provision of toys to children may be a fit subject for ‘charity’, but not meeting essential health needs.
**Pogge**	Does Pogge’s analysis of harm cohere with ordinary usage? Does it satisfy the description of a negative duty (i.e. an injunction to refrain from doing something, in this case, causing harm)?	Harm is always properly judged in relation to a subjunctive standard (i.e., the possibility of an alternative institutional order in which fewer serious harms are committed).
	Is Pogge’s empirical description of the global order accurate? Local factors such as poor governance or corruption are important in explaining the poverty of developing countries.	Pogge emphasises that local and global factors often interact in complex ways, and that local factors may often have current or past non-local causes [25]. While it may often be sufficient to point either to local causes or to global causes to explain the persistence a phenomenon such as severe poverty or poor health status, this recognition cannot diminish the share of moral responsibility attributable to either set of factors [22].
**Shue**	Shue’s concept of subsistence rights is indeterminate and may open the door to unduly extensive obligations	The concept of subsistence rights is not designed to foster global economic equality and is sufficiently clear to guide foreign policy.
**Rawls**	Individuals may be poorly served by a theory addressed primarily to peoples. One’s nation of birth is a matter of luck rather than choice, and is hence morally arbitrary. It should not influence life chances unduly. In addition, citizens may not be well represented by their head of state. We have stronger duties towards individuals than Rawls’s theory suggests.	If we address our theory to individuals rather than peoples, we risk undue interference in the domestic affairs of independent peoples and exceed the proper scope of justice.
	Is the thesis of explanatory nationalism, which holds that the key ingredient in how a country fares is its own political culture and traditions, correct?	Depends on one’s interpretation of empirical evidence.

^1^ These are criticisms commonly raised in the philosophical literature and by no means represent an exhaustive list. Rejoinders presented are consistent with the authors’ standpoint.

^2^ A general criticism of all consequentialist approaches would be that factors other than consequences are relevant to determining moral duties. Singer, like other consequentialists, would disagree.

**Table 3:** **Examples of policies that cohere with each of the four accounts of justice**^**1,2**^

**Policies**	**Singer**	**Pogge**	**Shue**	**Rawls**
Reform of international arrangements governing medical research and development^3^		X	X	X
Sustainable domestic policies for high-income countries in relation to human resources for health^4^		X	X	
Proportional compensation for the health effects of environmental pollution & climate change		X	X	X
Ensuring transparency and coherence in the effects of foreign and domestic policies on health worldwide		X	X	
Reducing inequalities in health between countries through foreign and domestic policies				
Reducing agricultural trade subsidies & other protectionist practices		X	X	X
Regulatory measures to contain speculation in financial and commodity markets		X		
Meeting financial commitments to global development initiatives, such as 0.7% GDP	X	X	X	
Support for the health-related MDGs	X	X	X	X
Support for the Global Fund to Fight AIDS, Tuberculosis and Malaria (GFATM)	X	X	X	X
Support for the Global Alliance for Vaccines and Immunisation (GAVI)	X	X	X	X
Support for the UN Global Strategy for Women’s and Children’s Health	X	X	X	X

^1^ Several of these policies were drawn from the UK “Health is global” report [19].

^2^ An “X” indicates that the policy would be supported. Detailed reasons are provided in the Additional file 2. Absence of an “X” means either that the answer is indeterminate (the theory is silent on these points) or negative.

^3^ Examples include the trade-related aspects of intellectual property rights (TRIPS) agreement, and so-called “TRIPS plus” bilateral agreements.

^4^ Specifically, ceasing to underfund medical training at the domestic level and to import qualified professionals from the developing world.

Each theory will take a position on the question of whether duties towards the health of those outside our borders are matters of “justice” or “charity”. Duties of justice are precise, owed to specifiable others, and should in principle be legally enforceable, whereas duties of charity admit of discretion in relation to their nature, timing, and choice of beneficiary. Such obligations are not legally enforceable.

### Peter Singer and the requirement to aid others in need

Princeton University philosopher Peter Singer writes from the perspective of consequentialism, a family of theories whose unifying element is a focus on outcomes. Consequentialists believe that consideration of outcomes forms the relevant basis for deciding which policies and practices are morally correct. Approaches differ in terms of the types of consequences taken to matter most. Some versions may specify a single good, such as pleasure or the avoidance of pain [26], while others promote the satisfaction of preferences [27], or an objective list of several goods to be promoted equally. Most forms of consequentialism focus on maximising beneficial outcomes, but this is not always the case.

Singer’s argument about our obligations to others is general, simple and, if true, profound. For Singer, every human being has the capacity for suffering and enjoyment or happiness, and is thus deserving of equal consideration [27]. Contrasting the estimated 8.8 million child deaths worldwide in 2008 due to preventable, poverty-related causes [28] with the relative comfort in which almost 1 billion people live, Singer maintains that the global rich have an obligation to alleviate the suffering of the global poor. He argues that, if we can prevent something importantly bad without sacrificing anything of comparable significance, we ought to do so. As the morbidity and premature death linked to extreme poverty is deeply bad and a significant proportion can be prevented without undue sacrifice, this ought to be done [24].

Singer’s theory addresses itself to individuals and asks that each individual moral agent give the same weight to the interests of others as to his or her own. For Singer, the moral point of view is inherently radically impartial, surmounting specific attachments to individuals, communities and countries.

### Thomas Pogge on global institutions and the duty not to harm

Asking why severe poverty and inequality persist worldwide, Yale University’s Thomas Pogge focuses on structural causes. Pogge asks whether the current global institutional order—for which the governments of the rich nations (and hence their citizens) bear primary responsibility— figures as a substantial contributor to the life-threatening poverty suffered by billions in the developing world [22].

Pogge challenges us to reflect on the relationship between the persistence of severe poverty and inequality worldwide and recent decisions concerning our path of globalization [22]. While the legacy of colonialism persists, Pogge’s argument focuses primarily on events since roughly 1980. He raises two issues: first, the governments of wealthy nations “enjoy a crushing advantage in terms of bargaining power and expertise;” and second, international negotiations are based on an adversarial system in which country level representatives seek to advance the best interests of their nation. Systematic consideration of the needs of the global poor is not a part of the mandate of any of the powerful parties to the negotiation. The cumulative results are, in Pogge’s view, predictable: a grossly unfair global order in which benefits flow predominantly to the affluent [22].

What effect do these asymmetries have on the health of those in developing countries? First, decisions taken by global institutions, state actors or corporations may cause or aggravate problems in securing critical determinants of health. While severe poverty is arguably most important, climate change and environmental damage also affect health determinants such as air, water and food. Negative consequences disproportionately impact the global poor, while the benefits of development have fallen mainly to the affluent. Second, decisions have at times impeded the ability of developing country governments to provide health care to their own citizens, for example through structural adjustment or trade policies. For Pogge, a particularly important issue concerns essential medicines [29]. He believes that the global medical innovation system embodied in the World Trade Organization (WTO)’s Trade Related Intellectual Property Rights (TRIPS) agreement is unjust. An independent commission confirmed that the benefits of the current system flow disproportionately towards rich countries [30].

Pogge invokes a central element of Western morality: it is wrong severely to harm innocent people for minor gains. The duty not to harm (a so-called negative duty, as distinct from positive duties like those to render assistance) is considered a strict obligation applicable equally to fellow citizens and foreigners. If Pogge is correct about the harm caused by our global institutions, this implies that we have an immediate duty of justice to those harmed regardless of where they live [22].

There has been much debate about Pogge’s proposal and the correct baseline for determining harm. Taking a “state of nature” perspective one might perhaps argue that, in the absence of something like the current global order, the global poor would have been no worse off.

This objection misconstrues Pogge’s claim. Pogge proposes that we appeal to human rights as a minimum standard for judging the adequacy of institutions. Inspired by the 1948 Universal Declaration of Human Rights which states: “Everyone is entitled to a social and international order in which the rights and freedoms set forth in this Declaration can be fully realized,” [31] he argues that any justifiable international order must be designed insofar as reasonably possible to guarantee human rights including basic freedoms of participation, subsistence, education and health care. Pogge argues that the attribution of harm implicitly involves a “subjunctive” (as opposed to an historical) comparison, and that the correct subjunctive comparison would be the possibility of a feasible alternative institutional order in which fewer human rights deficits would be produced [21,22,25].

In sum, for Pogge, a set of global institutional arrangements is unjust if it foreseeably perpetuates large-scale human rights deficits that could reasonably be avoided through feasible institutional modifications. He amasses empirical evidence to demonstrate that the citizens of wealthy nations via their elected governments contribute to the perpetuation of global poverty and ill health. If Pogge’s analysis is correct, we have a strict obligation of justice, grounded in the duty not to cause harm, to change our institutions and take concrete compensatory actions [22].

### Henry Shue on “basic rights”

Oxford University’s Henry Shue focusses on the role of human rights, especially economic rights, in international affairs. Discussions of human rights in the West have generally distinguished “civil and political” from “social, economic and cultural” rights and given priority to the former. Shue argues that the most fundamental core of the economic rights, which he calls “subsistence rights,” ought also to receive priority [23].

Shue maintains that there are basic rights to security and subsistence. His defence of subsistence as a basic right has three main components.

(1) Some charge that the right to subsistence is a “positive right” and thus inherently of lower priority. According to a commonly held liberal view, positive rights entail correlative duties to act, whereas negative rights entail duties merely not to violate and not to interfere with other’s fundamental freedoms. For example, the (negative) right to physical security can be understood as a right held by all implying a universal injunction to refrain from threatening the physical integrity of others. On this view, negative rights represent obligations for which one has a right to compel performance and impose sanctions for non-performance. Positive rights are more indeterminate; moreover, failure to comply confers no legal sanction. Shue counters this charge noting that all rights are in fact *mixed* and require both negative and positive actions to secure their enjoyment. For instance, the right to physical security implies not only that all citizens within a state refrain from assaulting one another, but also that the government undertake substantive steps to sustain a coercive system of justice and a police force.

(2) The right to physical integrity is often argued to have special priority in that no one can fully enjoy any right if her physical integrity is threatened. Shue makes a parallel case for subsistence rights. He argues that the rights to physical integrity and subsistence collectively provide the material preconditions necessary to the enjoyment of all other rights, such as the right to property, the right to equal political participation, and the right to freedom of association.

(3) To complement the idea of basic rights, Shue offers a theory of related duties. Essentially, “basic rights are everyone’s minimum reasonable demands upon the rest of humanity”; they call for three kinds of duties incumbent upon individuals and societies. These are: 1) the duty to avoid depriving; 2) the duty to protect from deprivation; and 3) the duty to aid the deprived.

What does Shue’s thesis about “basic rights” imply about transnational duties towards health? His response is somewhat ambivalent and falls short of asserting universal duties towards all those deprived of their basic rights. A particularly important challenge comes from an interlocutor who accepts the notion of universal subsistence rights, but argues that responsibility for their fulfilment rests with the nation of the bearer of the duty [23]. For Shue, duties beyond borders figure principally as “a back-up arrangement for the failure of so-called national governments” and come into play “where the state with the primary duty to protect rights fails - for lack of will or lack of capacity - to fulfill its duty”.[23] In essence, to the extent that liberal democracies accept that basic rights are fundamental to domestic justice, Shue argues that a principle of consistency requires that they also respect and promote basic rights through foreign policy in countries where appropriate institutional provisions are absent or incomplete. Therefore, even if national boundaries legitimately delimit political communities whose members share strong ties and obligations, states espousing liberal democratic values have a duty to adopt foreign policies consistent with basic rights.

The right to subsistence aims to guarantee every human being worldwide a decent chance at living a long and healthy life, and includes protection from extreme poverty and guarantees related to the social determinants of health, as well as elementary health care [23].

### John Rawls and the duty of assistance

Perhaps the most influential analyst of international responsibilities from a liberal perspective, the late Harvard philosopher John Rawls addressed the question of how reasonable citizens and peoples might live together peacefully in a just world. His work is animated by the belief that the greatest evils of human history—including war, persecution, starvation and poverty—are the consequence of *political* injustice, and the removal of such injustice the key to their resolution [32]. For Rawls, the fundamental subjects of international law are political societies or “peoples”, collective entities with specific concepts of right and justice whose territory is bounded by borders. The diversity of values and cultures among peoples is the result of legitimate free exercise of human reason, and tolerance requires that we refrain from imposition of a supposedly universal conception of human rights and liberal democracy at the international level.

Rawls’s description of a just international community is based on his description of justice at the national level [33]. Speaking of modern constitutional democracies, Rawls argues that a just state must structure economic opportunities and social conditions so as to guarantee “fair equality of opportunity” in terms of life chances of the members of different sectors of society. Within a framework of guaranteed rights and liberties, Rawls proposes that social and economic inequalities be permitted only to the extent that they are of greatest benefit to the least advantaged. He argues that these principles of social cooperation reflect the notion of “reciprocity,” or what it would be reasonable for free and equal persons ignorant of their specific future roles to accept in an ideal form of social contract [33].

At the international level Rawls envisages a similar hypothetical social contract. The representatives of peoples come together in a context of reciprocity, characterised by symmetry, freedom and equality of the parties. In a situation that masks specific knowledge of features such as country size, wealth and history, Rawls claims that the representatives would define eight principles of mutual governance, including a duty to “assist other peoples living under unfavourable conditions that prevent their having a just or decent political and social regime” [32].

#### The duty to aid burdened societies

Rawls distinguishes duties and norms of conduct governing the relationship of “well-ordered peoples” (generally, liberal democracies) to two types of societies: “outlaw states” that refuse to comply with international law, and—the focus of our interest—“burdened societies.” Rawls defines burdened societies as those that suffer from unfavourable circumstances that preclude them from developing just *political* institutions. Moreover, he maintains that the key element in how a country fares overall is its own political culture and traditions, rather than poor luck in its share of natural resources or external factors related to interactions between states [32]. This thesis, known as “explanatory nationalism,” is highly contested. In keeping with this view, Rawls limits universally valid human rights to political rights.

Although his eight rules of governance do not include a principle of distributive justice, Rawls holds that well-ordered societies have an important duty to assist burdened societies. He offers three points of guidance. First, mechanisms for assistance should be chosen so as to effect a change in the political culture and institutions of the burdened society. Rawls argues that economic transfers may not be most appropriate for realising this goal [32]. Among recommended courses of action, Rawls stresses the importance of policies and interventions that emphasise human rights, particularly those that further the rights and fundamental interests of women [32]. Second, while recognising that poverty and a lack of material resources may impact on a country’s ability to develop and maintain positive political institutions, the aim of the duty of assistance is not to compensate for material lacks, to equalise levels of wealth across societies, or to permit continuous economic growth. Third, the objective of assistance is to enable burdened societies to achieve just political arrangements. When this is achieved further assistance is not required, even if the society remains relatively poor [32].

#### Health & the duty of assistance

The aim of Rawls’ duty of assistance is to enable burdened societies to achieve just political arrangements. As this duty is framed in political terms it entails no obvious health-related obligations. Candidate strategies must be justified by demonstrating their contribution to just political arrangements. We argue that supporters of a rawlsian position should privilege health-related interventions, as empirical evidence shows that interventions to improve global health make an essential contribution to achieving just political arrangements. We offer two complementary reasons*.*

##### Unhealthy societies cannot be politically just

Rawls describes several criteria that must be satisfied in order for a society to be just. At the domestic level, a just society must satisfy Rawls’s principle of equality of opportunity [33]. Yet, there is extensive empirical evidence that health problems are disproportionately concentrated in disadvantaged population sub-groups, reflecting and exacerbating social and economic differences between the members of a society [34]. Everywhere the burden of disease is high, the chance to survive to adulthood, when the rights and privileges of democratic citizenship can be exercised, differs sharply across social groups. Deeply unhealthy societies therefore cannot guarantee that those with similar abilities, skills and initiative have similar life chances, regardless of starting point.

Out of respect for national sovereignty, Rawls offers a less stringent version of the equality of opportunity principle for state members of the just international community. The international version stipulates that all states must, at a minimum, maintain equality of opportunity in education and training [32]. However, child survival, school performance and life prospects are importantly affected by preventable and treatable health conditions, and negative effects are concentrated among vulnerable population sub-groups [3]. Where the burden of disease is high, the principle of equality of opportunity in education and training cannot be met.

Rawls also views basic economic entitlements as essential to just political arrangements [32]. A high burden of disease contributes to the entrenchment of poverty and threatens subsistence rights, with greatest impact upon the vulnerable and powerless [34,35]. For this ensemble of reasons, societies with a high burden of disease necessarily fail to meet criteria for just political arrangements.

##### Health interventions are a particularly effective way to promote just political arrangements

Conversely, for many otherwise vibrantly democratic developing nations, failure to achieve a reasonable standard of population health is a major impediment to achieving just political arrangements. Where the burden of disease is still high, improvements in population health would speed the process of transition to just societies by making it possible for individuals to enjoy real exercise of their rights, liberties and opportunities and to avoid destitution. Such policies would disproportionately promote the well-being and empowerment of women and children. Health interventions are also potentially very effective in stimulating sustainable economic growth and alleviating poverty [2].

## Discussion

The moral significance of national borders is perhaps the central question facing contemporary theories of justice. Noting that one’s country of birth is a matter of moral luck, cosmopolitan philosophers [22,24] argue that the deep inequalities that characterise our globe are injustices that ought to be corrected by the international community. Their nationalist counterparts [23,32] argue that the concept of justice does not properly apply in the international context. These philosophers highlight the absence of legitimate institutions of common governance at the global level and the importance of preserving national autonomy.

We have reviewed four theories taking different positions in this debate and highlighted the reasons that each might give to support initiatives to improve the health of the worst off worldwide [Table 1]. The four theories offer distinct rationales for intervention and suggest different limits on responsibilities, with cosmopolitan theories (Singer, Pogge) generally upholding more widespread and urgent responsibilities for health beyond borders than their nationalist counterparts (Shue, Rawls), who seek to qualify the scope of such duties. Notwithstanding, some important commonalities emerge.

First, whether conceived as obligations of justice or charity, responsibilities to provide international assistance are significant for all four theories [Table 1]. Even those theorists who see national borders as highly morally salient recognise the importance of some supranational obligations, in contradiction to the popular presumption that domestic concerns always have priority. In other words, there are limits to the scope of acceptable national autonomy.

Second, among the range of potential aid foci, interventions for health enjoy consistent prominence [Table 1. This reflects the inherent importance of health to individuals and its contribution to leading a dignified and fulfilling life [36], as well as the intimate link between health and development [2]. The importance of global health is explicit for Singer, Pogge and Shue, while for Rawls it follows from the effectiveness of health interventions in strengthening equality of opportunity and thereby, just political arrangements.

Third, despite significant theoretical disagreements [Tables 1, and 2, many of the most important current initiatives to promote global health can be supported by all four views [Table 3Additional file  2. An “overlapping consensus”[18] at the level of policy can thus be upheld from a variety of moral perspectives and by way of diverging views about the importance of national borders.

Our analysis has two important limitations. First, as this argument was developed through a review of the work of four contemporary philosophers, our conclusions reflect the frameworks selected for inclusion and the specific interpretations given these theories. Our selection of theories was careful and purposive, and we believe that they do represent the most important viewpoints in contemporary discussions of justice. Moreover, although limitations of space prevent us from undertaking a demonstration, we believe that the overwhelming majority of contemporary theories of justice could support a similar justification for action on global health. While we acknowledge the existence of viewpoints that might not support our conclusions, we wish to underscore the remarkable degree of support for current global health interventions among prominent competing frameworks.

Second, given the inherently controversial nature of ethical choices, a separate challenge relates to the value of pursuing a normative approach. One might ask, would it not be preferable to base the argument on pragmatic reasons for action such as enlightened self-interest, or protection of common interests? Pragmatic reasons offer extremely important sources of motivation in many instances. However, our self-interest is not always served by doing what is right. The current global situation has clear winners and losers. To the extent that the contemporary state of global health reflects “a toxic combination of poor social policies and programmes, unfair economic arrangements, and bad politics” [37], the remedy cannot come from the powerless.

The MDGs represent a landmark pledge of solidarity on the part of the international community towards the global poor. As the target date for their fulfilment approaches, recent crises related to instability in financial markets and in food and commodity prices, as well as environmental change, threaten to undermine hard-won gains in health and prosperity while jeopardising future availability of overseas development assistance (ODA). ODA is only one of many policy channels affecting global health and development [38]; however, it plays a crucial role [8,37]. Choices made by the citizens and governments of the wealthy nations in the next short while will be particularly decisive. The overlapping normative consensus we have identified in favour of action on global health is undoubtedly fragile; yet, it resonates with the broad based public support enjoyed by key global health initiatives. We are hopeful that an informed dialogue on ethics can enable individuals and governments to find a more reasoned basis for their views. The most effective resource of the global poor may be a transformation of moral vision on the part of the powerful [22].

## Abbreviations

AIDS, Acquired Immune Deficiency Syndrome; DAH, International Development Assistance for Health; G8, Group of Eight Countries; HIV, Human Immunodeficiency Virus; MDGs, Millennium Development Goals; ODA, Overseas Development Assistance; TRIPS, Trade Related Intellectual Property Rights; WTO, World Trade Organization.

## Competing interests

The authors declare that they have no competing interests, financial or otherwise, in completion of this work. MJ collaborates on a pro bono basis with the World Health Organization (WHO), the GAVI Alliance, and the Global Fund to Fight AIDS, Tuberculosis and Malaria (GFATM), and has received research funding from GFATM and WHO unrelated to this project. RC has no relationships to disclose. On a pro bono basis, AJD has collaborated with WHO in the past, and is a current member of the Research Ethics Board of Médecins Sans Frontières (MSF) and the Public and Political Support Working Group for the Decade of Vaccines Collaboration. He has received research support from the Bill and Melinda Gates Foundation unrelated to this project. TS coordinated the Globalization Knowledge Network of the WHO Commission on Social Determinants Of Health (CSDH) with funding from the International Affairs Directorate, Health Canada. The authors assume sole responsibility for the opinions expressed in this work.

## Authors’ contributions

MJ conceived the study and drafted the manuscript. RC and AJD contributed to conception and design of the study, drafting of the manuscript, and critical revision of the manuscript for important intellectual content. TS contributed to conception and design of the study and revision of the manuscript for important intellectual content. All authors have approved the final version.

## Supplementary Material

Additional file 1 Study Selection Description of data: describes process of study selection. Click here for file

Additional file 2 **(Expanded Table 3):** Provides examples of policies that cohere with each of the four accounts of justice. Click here for file
