# Supplementary material for: Global health and national borders: the ethics of foreign aid in a time of financial crisis
Source: Global Health. 2012 Jun 28;8:19. doi: 10.1186/1744-8603-8-19 (PMC3464702; doi:10.1186/1744-8603-8-19)
Supplement: Additional file 1 — Study Selection Description of data: describes process of study selection. [file 1744-8603-8-19-S1.doc]

# Additional file 1: Study Selection

Authors individually established an initial list of theories for inclusion in the review. Criteria for the assessment were proposed by MJ.

### A1. Initial list of 11 candidate theories identified by the authors

| *Authors***\Criteria^1^** | **i** | **ii** | **iii** | **iv** | Comments |
| --- | --- | --- | --- | --- | --- |
| Beitz, Charles | + |  |  | + | Secondary to Rawls |
| Miller, David | + |  |  | + | Secondary to Rawls |
| Nozick, Robert | + | + | + | + | Libertarian, argumentatively weak |
| Nussbaum, Martha | + |  |  |  | Nussbaum and Sen considered together |
| O’Neill, Onora | + |  | + | + | Kantian (deontological approach) |
| Pogge, Thomas | + | + | + | + | Relational approach & has written directly on global health; essential to include |
| Rawls, John | + | + | + | + | Social contract approach; most important philosopher of 20^th^ century, essential to include |
| Sen, Amartya | + | + | + |  | Does not thematise issues of national borders; of extraordinary importance, has been widely discussed in the health sphere |
| Shue, Henry | + | + | + | + | Human rights approach |
| Singer, Peter | + | + | + | + | Consequentialist approach; essential to include |
| Young, Iris Marion | + |  |  | + | Similar in some respects to Pogge; not directly about health |

**^1^**The four criteria are: (i) Articulate important and widely held moral intuitions, (ii) Have had extensive impact on debates about global justice, (iii) Represent diverse approaches to moral reasoning, (iv) Present distinct stances on the normative importance of national borders

A group discussion was held to refine judgements and come to a common list of theories. In discussion, concerns were raised over the fact that female authors were judged to be less influential than male authors, and that the list contained only authors who published in English. Table S1 presents the consensus assessment following group discussion including all 11 frameworks proposed. The list was next shortened from 11 (comprehensive list including all suggestions) to 6 theories (the maximum judged to be feasible to present in a single paper).

**A2. Revised list of 6 theories presented to the Canadian Conference on International Health (CCIH), 2009**

| *Authors***\Criteria^1^** | **i** | **ii** | **iii** | **iv** | Comments |
| --- | --- | --- | --- | --- | --- |
| O’Neill, Onora | + |  | + | + | Kantian (deontological approach) |
| Pogge, Thomas | + | + | + | + | Relational approach & has written directly on global health; essential to include |
| Rawls, John | + | + | + | + | Social contract approach; essential to include |
| Sen, Amartya | + | + | + |  | Does not thematise issues of national borders; has been widely discussed in the health sphere |
| Shue, Henry | + | + | + | + | Human rights approach |
| Singer, Peter | + | + | + | + | Consequentialist approach; essential to include |

**^1^**The four criteria are: (i) Articulate important and widely held moral intuitions, (ii) Have had extensive impact on debates about global justice, (iii) Represent diverse approaches to moral reasoning, (iv) Present distinct stances on the normative importance of national borders

Participants at a special panel on global health equity at the Canadian Conference on International Health (CCIH) in 2009 were asked to provide feedback on the approach. Reactions were very positive and the approach was described as compelling and necessary; however, it was suggested that we reduce the number of frameworks to four. We followed this suggestion and eliminated Amartya Sen and Onora O’Neill. Sen was eliminated because his work is already well known to specialists in health and because he does not take a clear position on the issue of national borders. We regretted excluding O’Neill as she was the only deontologist and the only female writer; however, the other authors were judged more essential.

**A3. Final list of 4 theories presented to the International Studies Association (ISA), 2011**

| *Authors***\Criteria^1^** | **i** | **ii** | **iii** | **iv** | Comments |
| --- | --- | --- | --- | --- | --- |
| Pogge, Thomas | + | + | + | + | Relational approach & has written directly on global health; essential to include |
| Rawls, John | + | + | + | + | Social contract approach; essential to include |
| Shue, Henry | + | + | + | + | Human rights approach |
| Singer, Peter | + | + | + | + | Consequentialist approach; essential to include |

**^1^**The four criteria are: (i) Articulate important and widely held moral intuitions, (ii) Have had extensive impact on debates about global justice, (iii) Represent diverse approaches to moral reasoning, (iv) Present distinct stances on the normative importance of national borders

Participants at an invitation-only workshop on Health and Justice at the International Studies Association (ISA) 2011 Annual Convention “Global Governance: Political Authority In Transition” were asked to comment on criteria and choice of authors. The roughly twenty participants were internationally recognised experts in ethics and political philosophy, or in public health. The ISA panellists felt that criteria were reasonable and that the analysis was useful. They had some suggestions in terms of the selection of representative authors. To advance diversity (criterion iii), they felt that it might have been useful to consider the work of a more radical nationalist than Rawls such as David Miller. However, in terms of impact (criterion ii) Rawls was universally seen to be more important. After discussion, commentators agreed that David Miller would also support the claim that there are some global moral responsibilities for health as attested by his recent work in *National Responsibility and Global Justice*, Oxford University Press, 2007. To advance diversity (criterion iii), they also suggested consideration of libertarianism. In discussion, the group could not identify an appropriate proponent with a cogent philosophical perspective, nor did they feel that its importance was sufficient to warrant sacrificing any of the other perspectives (in essence, they felt that criteria i and ii told against inclusion). Although we had not shared the original list of candidate theories with ISA, the panellists suggested no frameworks or authors that were not on the original comprehensive list S1.
