# Supplementary material for: Global health and national borders: the ethics of foreign aid in a time of financial crisis
Source: Global Health. 2012 Jun 28;8:19. doi: 10.1186/1744-8603-8-19 (PMC3464702; doi:10.1186/1744-8603-8-19)
Supplement: Additional file 2 — (Expanded Table 3): Provides examples of policies that cohere with each of the four accounts of justice. [file 1744-8603-8-19-S2.doc]

**Additional File 2 (Expanded Table 3): Examples of policies that cohere with each of the four accounts of justice [22,38]**

| **Policies** | **Singer** | **Pogge** | **Shue** | **Rawls** |
| --- | --- | --- | --- | --- |
| Reform of international arrangements governing medical research and development [31] |  | X | X | X |
| Singer's theory is silent on these issues, but the general approach is likely to be supportive | | | | |
| Pogge explicitly analyses the effects of these arrangements and offers proposals for their reform. See, for example [38] | | | | |
| Shue does not explicitly consider these issues. However, he argues explicitly in favour of the concept that subsistence rights imply correlative duties, including the duty to promote policy coherence so as to avoid contributing to deprivations abroad. See: [22] p. 55-65 | | | | |
| Rawls does not explicitly consider these issues, but he believes that the community of ideal states must guarantee the fairness of market transactions by guarding against monopolitistic, oligopolistic or cartel- like behaviours by the more powerful economies [31] (p.43). It must ensure that unjustified inequalities among people do not develop over time, and correct those that do occur via the duty of assistance. [31] (p.43). To the extent that one sees the current medical innovation system as exhibiting features of monopolitistic, oligopolistic or cartel- like behaviours favouring the more powerful economies, Rawls would therefore favour reform and provision of compensation. | | | | |
| Sustainable domestic policies for high-income countries in relation to human resources for health [21] |  | X | X |  |
| Singer's theory is silent on these issues, but the general approach is likely to be supportive | | | | |
| Pogge does not explicitly consider this issue, but his work centres precisely on moral responsibilities for the harms caused by these kinds of institutional arrangements. See: [21] especially pp.112-116. | | | | |
| Shue does not explicitly consider these issues. However, he argues explicitly in favour of the concept that subsistence rights imply correlative duties, including the duty to promote policy coherence so as to avoid contributing to deprivations abroad. See: [22] p. 55-65 | | | | |
| Rawls does not explicitly consider these issues. This behaviour does not seem to be properly described as monopolitistic, oligopolistic or cartel- like behaviours on the part of the wealthier economies. It is hence not likely that he would see it as contravening the Law of Peoples. However, he sees the problem of immigration as a response to non-ideal conditions that would be eliminated once the ideal society of peoples is achieved. See [31] pp. 8-9. | | | | |
| Proportional compensation for the health effects of environmental pollution & climate change |  | X | X | X |
| Singer's theory is silent on these issues, but the general approach is likely to be supportive | | | | |
| Pogge does not explicitly consider this issue, but his work centres precisely on moral responsibilities for the harms caused by these kinds of institutional arrangements. See:^21^ especially pp.112-116 and the discussion of a global resources dividend pp. 196-215. On the concept of proportional compensation, see. [20] | | | | |
| Shue does not explicitly consider these issues. However, he argues explicitly in favour of the concept that subsistence rights imply correlative duties, including the duty to promote policy coherence so as to avoid contributing to deprivations abroad. See: [22] p. 55-65 | | | | |
| Rawls does not consider the health effects of environmental pollution and climate change, but he does hold that peoples have a responsibility to properly shepherd domestic environmental resources to enable their societies to maintain themselves in perpetuity. Failure to properly shepherd environmental resources leads to problems of sustainability of societies over time and contravenes the spirit of the Law of Peoples. See [31] pp. 8, 38-39. | | | | |
| Ensuring transparency and coherence in the effects of foreign and domestic policies on health worldwide |  | X | X |  |
| Singer's theory is silent on these issues, but the general approach is likely to be supportive | | | | |
| Pogge’s work explicitly draws attention to the need for transparency and policy coherence. For a discussion of moral responsibilities for the health effects of institutional arrangements, see [24] | | | | |
| Shue argues explicitly in favour of the concept that subsistence rights imply correlative duties, including the duty to promote policy coherence so as to avoid contributing to deprivations abroad. See: [22] p. 55-65 | | | | |
| Rawls’s theory is silent on these issues | | | | |
| Reducing inequalities in health between countries through foreign and domestic policies |  |  |  |  |
| None of the authors casts his argument in terms of reducing inequalities *per se*. Rawls explicitly argues that reduction in economic inequalities is not an appropriate target of the duty to aid burdened societies. See^31^ p. 111. For Singer, all suffering is morally relevant, and inequalities in health will be relevant to the extent that they are indicative of suffering.^27^ For Pogge, where we are causally responsible for ill health, arguments of justice apply.^24^ For all four authors, where inequalities in health reflect particularly severe or life threatening conditions, important moral arguments will apply (see below). | | | | |
| Reducing agricultural trade subsidies & other protectionist practices |  | X | X | X |
| Singer's theory is silent on these issues, but the general approach is likely to be supportive | | | | |
| Pogge’s work centres precisely on moral responsibilities for the harms caused by these kinds of institutional arrangements. See:^21^ especially pp.112-116 | | | | |
| Shue argues explicitly in favour of the concept that subsistence rights imply correlative duties, including the duty to promote policy coherence in domestic and foreign policy so as to avoid contributing to deprivations abroad. See: [22] p. 55-65 Agricultural subsidies and other protectionist practices are matters of state policy. | | | | |
| Rawls does not explicitly consider these issues, but he believes that the community of ideal states must guarantee the fairness of market transactions by guarding against monopolitistic, oligopolistic or cartel- like behaviours by the more powerful economies [31] (p.43). It must ensure that unjustified inequalities among people do not develop over time, and correct those that do occur via the duty of assistance [31]. (p.43). To the extent that agricultural trade subsidies and similar practices represent monopolitistic, oligopolistic or cartel- like behaviours on the part of the more powerful economies and violate the fairness of market transactions, they should be reformed and compensation provided. | | | | |
| Regulatory measures to contain speculation in financial and commodity markets |  | X | ? |  |
| Singer's theory is silent on these issues, but the general approach is likely to be supportive | | | | |
| Pogge’s work centres precisely on moral responsibilities for the harms caused by these kinds of institutional arrangements. See: [21] especially pp.112-116 | | | | |
| Shue argues explicitly in favour of the concept that subsistence rights imply correlative duties, including the duty to promote policy coherence so as to avoid contributing to deprivations abroad. See: [22] p. 55-65 Introduction of regulatory measures is consistent with the spirit of his work, and he explicitly discusses the value of institutional reform to protect, promote and fulfil basic rights. See: [22] p.166-173. | | | | |
| Rawls’ theory is silent on these issues | | | | |
| Meeting financial commitments to global development initiatives, such as 0.7% GDP | X | X | X |  |
| Meeting such commitments would contribute to relieving extreme suffering [23]. | | | | |
| Meeting such commitments would contribute to fairer global institutional arrangements [21] [20]. | | | | |
| Meeting such commitments would contribute to realisation of basic rights [22]. | | | | |
| Rawls explicitly argues that reduction in economic inequalities is not an appropriate target of the duty to aid burdened societies. See [31] p. 111. The importance of meeting these financial commitments would hence depend on what kinds of strategies are envisaged. Rawls would favour only those strategies consistent with the duty to aid burdened societies. See [31] pp. 105-110. | | | | |
| Support for the health-related MDGs | X | X | X | X |
| Would contribute to relieving extreme suffering [23]. | | | | |
| Would contribute to realisation of human rights [21] [20]. | | | | |
| Would contribute to realisation of basic rights [22]. | | | | |
| Would contribute to realisation of just political arrangements through promotion of equality of opportunity and realisation of subsistence rights. These are necessary for meaningful enjoyment of democratic rights [31]. | | | | |
| Support for the Global Fund to Fight AIDS, Tuberculosis and Malaria (GFATM) | X | X | X | X |
| Would contribute to relieving extreme suffering [23]. | | | | |
| Would contribute to realisation of human rights [21] [20]. | | | | |
| Would contribute to realisation of basic rights [22]. | | | | |
| Would contribute to realisation of just political arrangements through promotion of equality of opportunity and realisation of subsistence rights [31]. GFATM plays a particularly important role in promoting the health and human rights of women and girls (HIV), and of children (malaria). While the importance of strategies that target women is highlighted by Rawls due to their role in development, the health of women and (especially) children is also key to promoting equality of opportunity. The HIV/AIDS epidemic is also a recognised threat to international security. Through its work on HIV/AIDS, the Global Fund also addresses threats to peace that the international community has prudential reasons to alleviate [31]. Peace, freedom from extreme poverty and equality of opportunity are essential to developing and maintaining just political institutions. | | | | |
| Support for the Global Alliance for Vaccines and Immunisation (GAVI Alliance) | X | X | X | X |
| Would contribute to relieving extreme suffering [23]. | | | | |
| Would contribute to realisation of human rights [21] [20]. | | | | |
| Would contribute to realisation of basic rights [22]. | | | | |
| Would contribute to realisation of just political arrangements through promotion of equality of opportunity and realisation of subsistence rights [31]. Although some antigens target adults, the GAVI Alliance plays a particularly important role in promotion of child health through vaccination of infants with existing, new and underused antigens. Child health is key to ensuring equality of opportunity. | | | | |
| Support for the UN Global Strategy for Women’s and Children’s Health | X | X | X | X |
| Would contribute to relieving extreme suffering [23]. | | | | |
| Would contribute to realisation of human rights [21] [20]. | | | | |
| Would contribute to realisation of basic rights [22]. | | | | |
| Would contribute to realisation of just political arrangements through promotion of equality of opportunity and realisation of subsistence rights [31]. While the importance of strategies that target women is highlighted by Rawls due to their role in development, the health of women and (especially) children is also key to promoting equality of opportunity. | | | | |

^1^ Several of these policies were drawn from the UK “Health is global” report [25].

^2^ An “X” indicates that the policy would be supported. Detailed reasons are provided in the accompanying Webtable. Absence of an “X” means either that the answer is indeterminate (the theory is silent on these points) or negative.

^3^ Examples include the trade-related aspects of intellectual property rights (TRIPS) agreement, and so-called “TRIPS plus” bilateral agreements

^4^ Specifically, ceasing to underfund medical training at the domestic level and to import qualified professionals from the developing world
